# Uterine choriocarcinoma arising from serous carcinoma in a postmenopausal woman: an analysis of next-generation sequencing and PD-L1 immunochemistry

**DOI:** 10.1186/s13000-022-01262-z

**Published:** 2022-10-13

**Authors:** Meiping Li, Lei Bao, Bo Lu, Wenshun Ge, Lifang Ren

**Affiliations:** Department of Surgical Pathology, Shaoxing Maternity and Child Health Care Hospital, East Street 305#, Shaoxing, Zhejiang Province 312000 China

**Keywords:** Choriocarcinoma, Endometrium, Serous carcinoma, PD-L1, Next-generation sequencing

## Abstract

**Background:**

Uterine somatic choriocarcinoma is a rare, clinically aggressive malignant tumor. They frequently concur with other cancer. However, the molecular pathogenesis between somatic choriocarcinoma and the concurrent carcinoma has rarely been addressed to date.

**Case presentation:**

We report a 68-years old Chinese woman with a uterine choriocarcinoma arising from serous carcinoma. The patient underwent radical surgery including total abdominal hysterectomy with bilateral salpingo-oophorectomy, omentectomy and pelvic lymph node resection. She received 10 courses of post-operative chemotherapy. She died of disease 13 months after her surgery. Microscopically, the tumor showed a biphasic pattern of choriocarcinoma and serous carcinoma. The choriocarcinomatous component showed a combination of cytotrophoblast, intermediate trophoblast and syncytiotrophoblast with hemorrhage and necrosis. The component of serous carcinoma was characterized by solid sheets of small cells with marked nuclear atypia and occasional glandular and papillary formation. PD-L1 was exclusively expressed in the choriocarcinomatous component. Next-generation sequencing revealed that the genetic abnormalities were overlapping between the two components.

## Background

Choriocarcinoma is a highly malignant tumor composed of neoplastic syncytiotrophoblast, intermediate trophoblast, and cytotrophoblast. It is usually gestational in origin. Non-gestational choriocarcinoma can be originated from germ cell or from a somatic high-grade malignant tumor [1]. Somatic choriocarcinoma is a rare entity with an aggressive clinical course. It has been hypothesized to be associated with aberrant differentiation within a somatic epithelial neoplasm although the molecular evidence remains very limited. In this manuscript, we report a case of uterine choriocarcinoma arising from serous carcinoma in a postmenopausal woman with detailed clinicopathological and molecular analysis.

## Case presentation

A 68-year-old, G2P2, postmenopausal Chinese woman was admitted to our hospital. She complained of vaginal bleeding for 10 days. Gynecological physical examination revealed no significant abnormalities. Abdominal CT showed an intrauterine mass. Serum HCG was 6374.21U/L. The pathological diagnosis of preoperative curettage was high grade endometrial carcinoma with choriocarcinomatous differentiation.

The patient underwent total abdominal hysterectomy with bilateral salpingo-oophorectomy, omentectomy, pelvic lymph node resection, and peritoneal multifocal biopsy. After surgery, the patient received 6 courses of paclitaxel and carboplatin (TC) initially, followed by 4 courses of actinomycin D and 5-Fu. However, her serum hCG remained abnormal continuously, up to 24867 IU/L after her last chemotherapy. She died of disease at 13 months after her surgery.

## Materials and methods

Immunohistochemical (IHC) staining was performed on formalin-fixed, paraffin-embedded tissue sections, using PT Link and Dako Autostainer Link 48 and REAL EnVision detection Kit (EnVision ™ FLEX + systems, DAKO). The antibodies used included: Estrogen Receptor (ER, clone: EP1), Progesterone Receptor (PR, clone: 1E2), Ki67 (clone: MIB-1), Paired box 8 (Pax8, clone: EP298), Insulin-like growth factor mRNA-binding protein 3 (IMP3, clone: EP286), P53 (clone: DO-7), Vimentin (clone: V9), Spalt-like transcription factor 4 (SALL4, clone: 6E3), Gata3 (clone: EP368), MLH1 (clone: ES05), PMS2 (clone: EP51), MSH2 (clone: RED2), MSH6 (clone: EP49), β-human chorionic Gonadotropin (β-HCG, clone: CG04 + CG05), Programmed cell death-Ligand 1 (PD-L1, clone: 22C3). All antibodies were prediluted (ready-to-use).

Genomic DNA was extracted from the two components with macro-dissection in 5-μm formalin-fixed paraffin-embedded (FFPE) tissue sections. Next-generation sequencing (1500 ×) was performed in the Geneseeq Laboratory, a College of American Pathologists (CAP)–accredited and Clinical Laboratory Improvement Amendments (CLIA)–certified laboratory (Geneseeq, Nanjing, China) using the GENESEEQ PRIME™ panel with probes targeting all exons of 437 cancer related genes, alternative splicing sites, selected introns with gene arrangement and microsatellite sites (Nanjing Shihe Gene Biotechnology Co. Ltd, China). The sequencing data were analyzed by a custom bioinformatics pipeline to detect various genomic variations.

### Pathologic findings

**Fig. 1:**
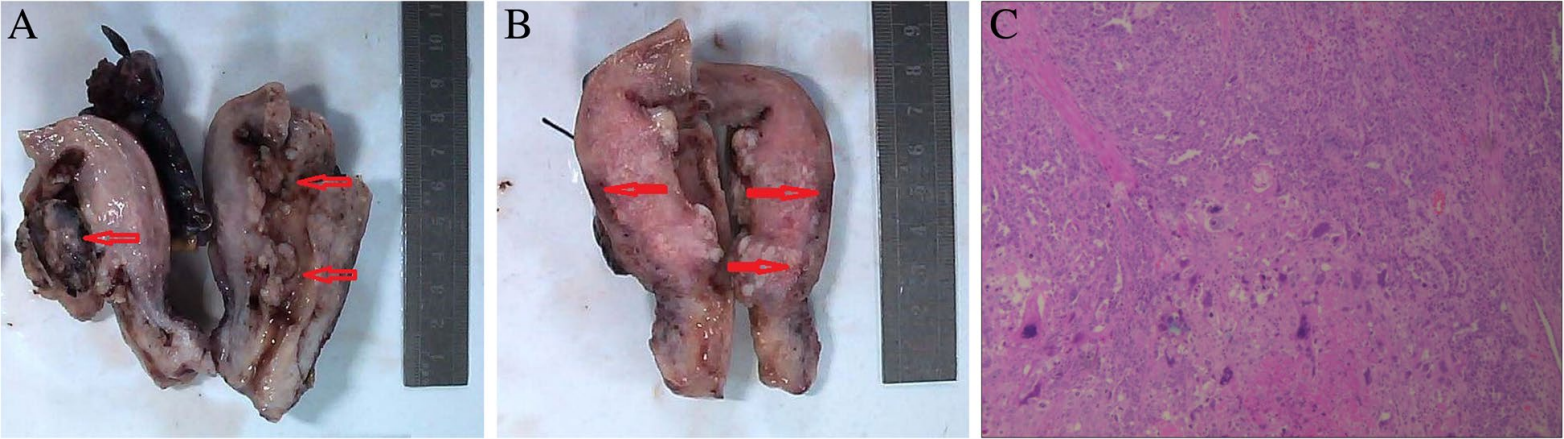
**A** Multiple nodular masses (the red arrow) with hemorrhage and necrosis were found in the uterine cavity **B**: The cut surface was pale gray and brown. The tumor penetrated the entire uterine wall (the red arrow) **C**: The tumor was consisted of serous carcinoma (upper) and choriocarcinoma (below). (Hematoxylin and eosin stain, original magnification, × 100)

Grossly, the uterus measured 9 cm × 6 cm × 3 cm. The serosal surface was smooth. The thickness of myometrium measured 2–2.3 cm. Th multiple masses in the uterine cavity measured 1.5-4 cm the diameters (Fig. 1A). The cut surface was gray and white. It frequently had areas with hemorrhage and necrosis. The tumor involved the entire uterine layers (Fig. 1B). The left ovary closely adhered with the fallopian tube. The dilated ampullae of bilateral fallopian tubes showed a solid, gray and yellow appearance, and contained multiple necrosis foci inside. The right ovary and fallopian tube looked unremarkable grossly.

**Fig. 2:**
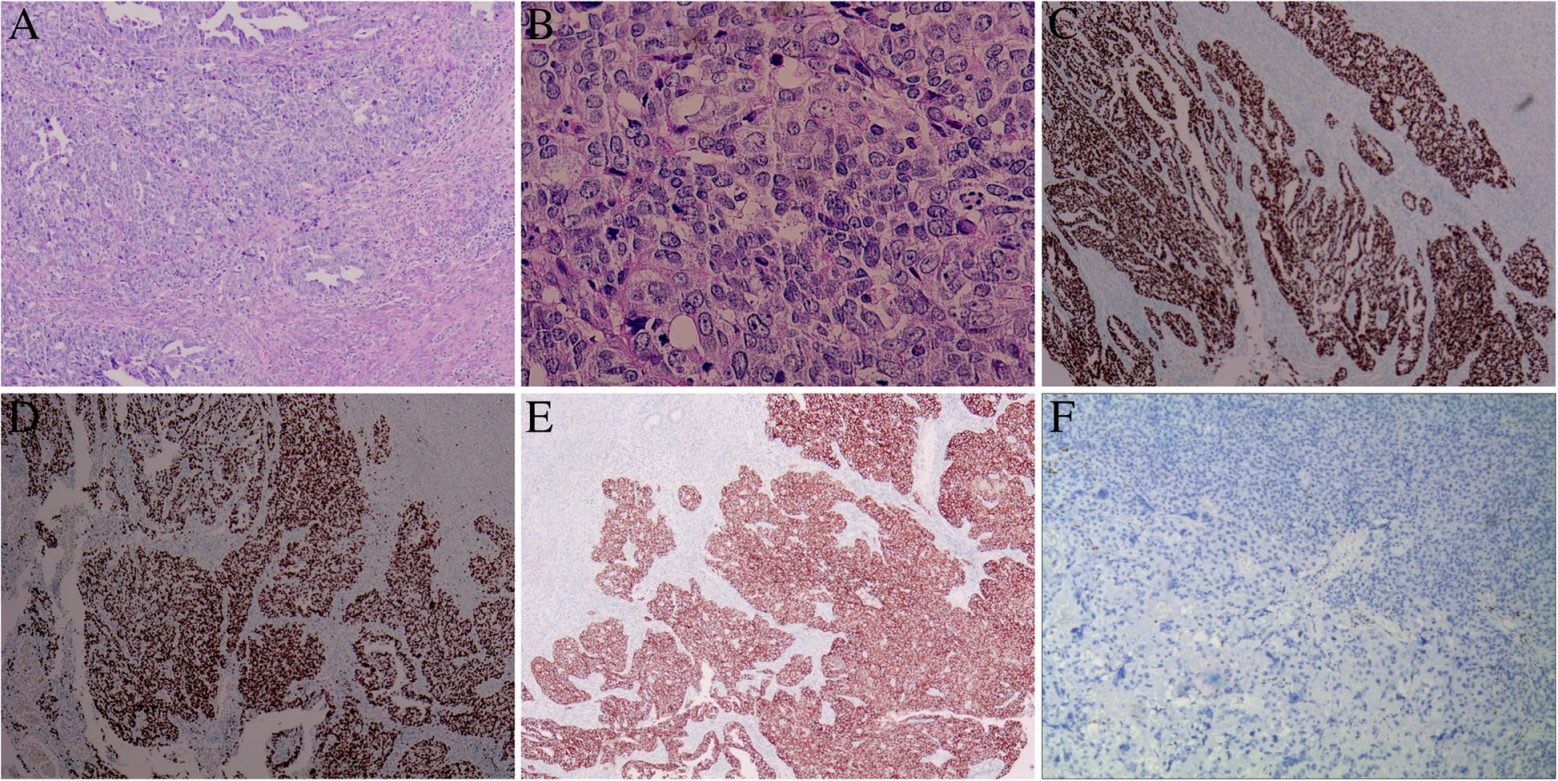
**A** Serous carcinoma showed glandular, papillary formation, and solid sheets. (Hematoxylin and eosin stain, original magnification, × 100) **B**: Serous carcinoma showed marked nuclear atypia, distinct nucleoli and frequent mitotic figures. (Hematoxylin and eosin stain, original magnification, × 400) **C**: Aberrant p53 over-expression in serous carcinoma. (The immunohistochemical stain, original magnification, × 50) **D**: High Ki67 index in srous carcinoma. (The immunohistochemical stain, original magnification, × 50) **E**: Positive IMP3 in srous carcinoma. (The immunohistochemical stain, original magnification, × 100) **F**: Negative ER in srous carcinoma. and CC. (The immunohistochemical stains, original magnification, × 100)

Microscopically, the tumor had two components, poorly-differentiated carcinoma and choriocarcinoma (Fig. 1C).The carcinomatous component accounted for 30–35%. It arranged in solid sheets predominantly, and in confluent glands and papillae occasionally. The tumor cells were small to moderate in size. They showed significant atypia. The larger, hyperchromatic nuclei had small but distinct nucleolus. The mitotic figures were frequently (> 10/10HPFs). The cytoplasm is light stained (Fig. 2A-B). The adjacent endometrium was unremarkable. The tumor cells showed a p53 mutant overexpression pattern (Fig. 2C), a high Ki67 index (~ 80%) (Fig. 2D), positive IMP3 (Fig. 2E) and Pax8, and negative ER (Fig. 2F) by IHC staining. The high grade carcinoma was consistent with an endometrial serous carcinoma (ESC).

**Fig. 3:**
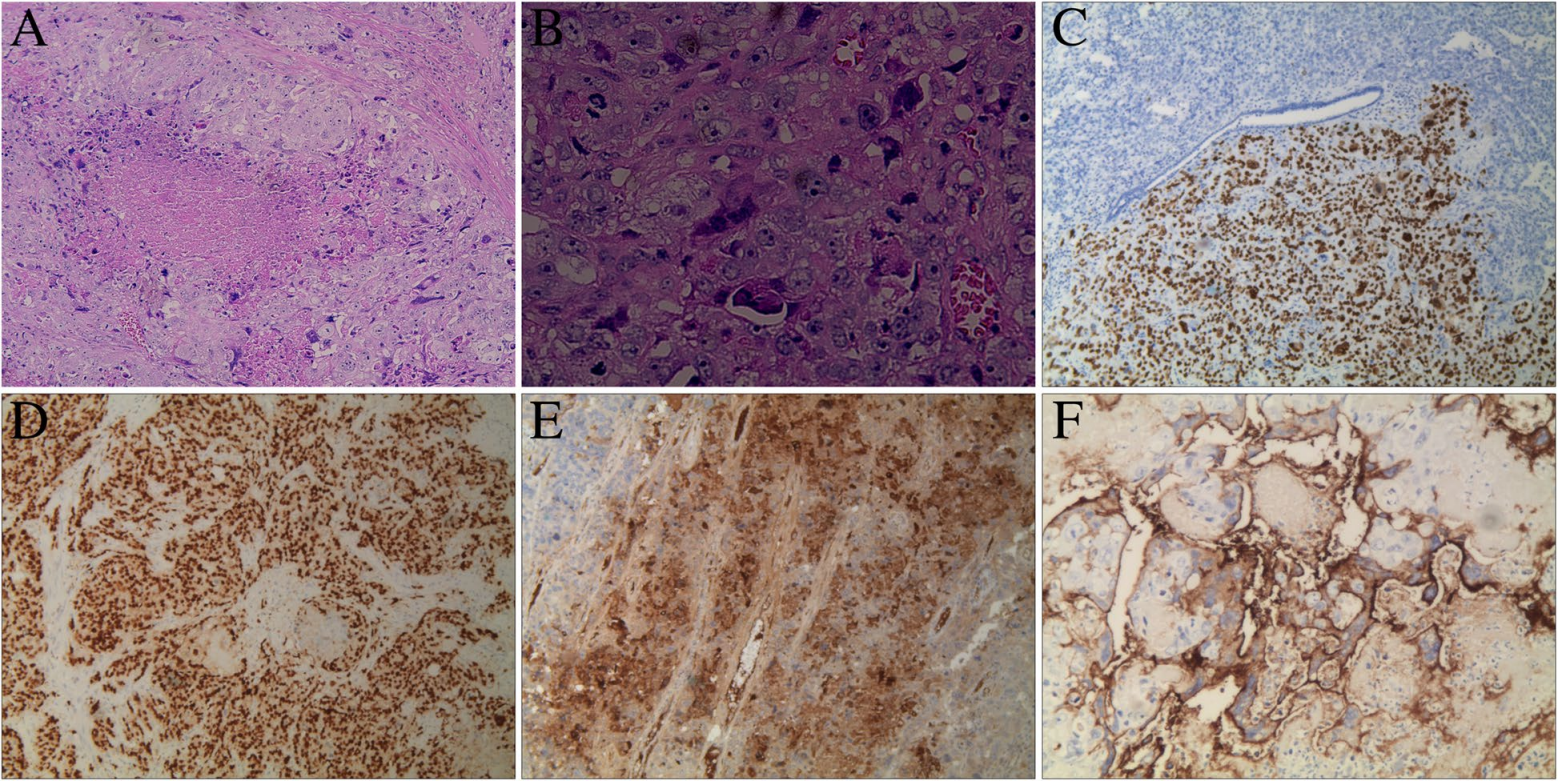
**A** Choriocarcinoma was consisted of mononucleate and multinucleate trophoblastic cell accompanied by extensive necrosis. (Hematoxylin and eosin stain, original magnification, × 100) **B**: Higher magnification of CC. (Hematoxylin and eosin stain, original magnification, × 400) **C**: Positive SALL4 in CC but negative in srous carcinoma. (The immunohistochemical stains, original magnification, × 100) **D**: Positive GATA3 in CC. (The immunohistochemical stains, original magnification, × 200) **E**: Positive HCG in CC. (The immunohistochemical stains, original magnification, × 200) **F**: PD-L1 positive in CC. (The immunohistochemical stain, original magnification, × 400)

The component of choriocarcinoma (CC) approximately accounted for 65–70%. It was composed of mononucleate and multinucleate trophoblastic cells with extensive hemorrhage and necrosis. It was characterized by a plexiform structure, in which, cytotrophoblast and intermediate trophoblasts were surrounded by syncytiotrophoblast. The significantly atypical trophoblasts showed hyperchromatic nuclei with obvious nucleoli and frequent mitotic figures. The cytoplasm was eosinophilic or transparent (Fig. 3A-B). CC was positive for SALL4 (Fig. 3C), GATA3 (Fig. 3D), HCG (Fig. 3E), and PD-L1 (Fig. 3F), and was negative for ER, PR, Pax8 by immunohistochemistry.

The tumor penetrated the whole uterine and invaded the wall rectal serosa and left pelvic lateral wall. Lymph-vascular space involvement (LVSI) was frequently present. The omentum and pelvic lymph nodes were free of tumor. The FIGO stage was IIIA.

### Next generation sequencing

**Fig. 4:**
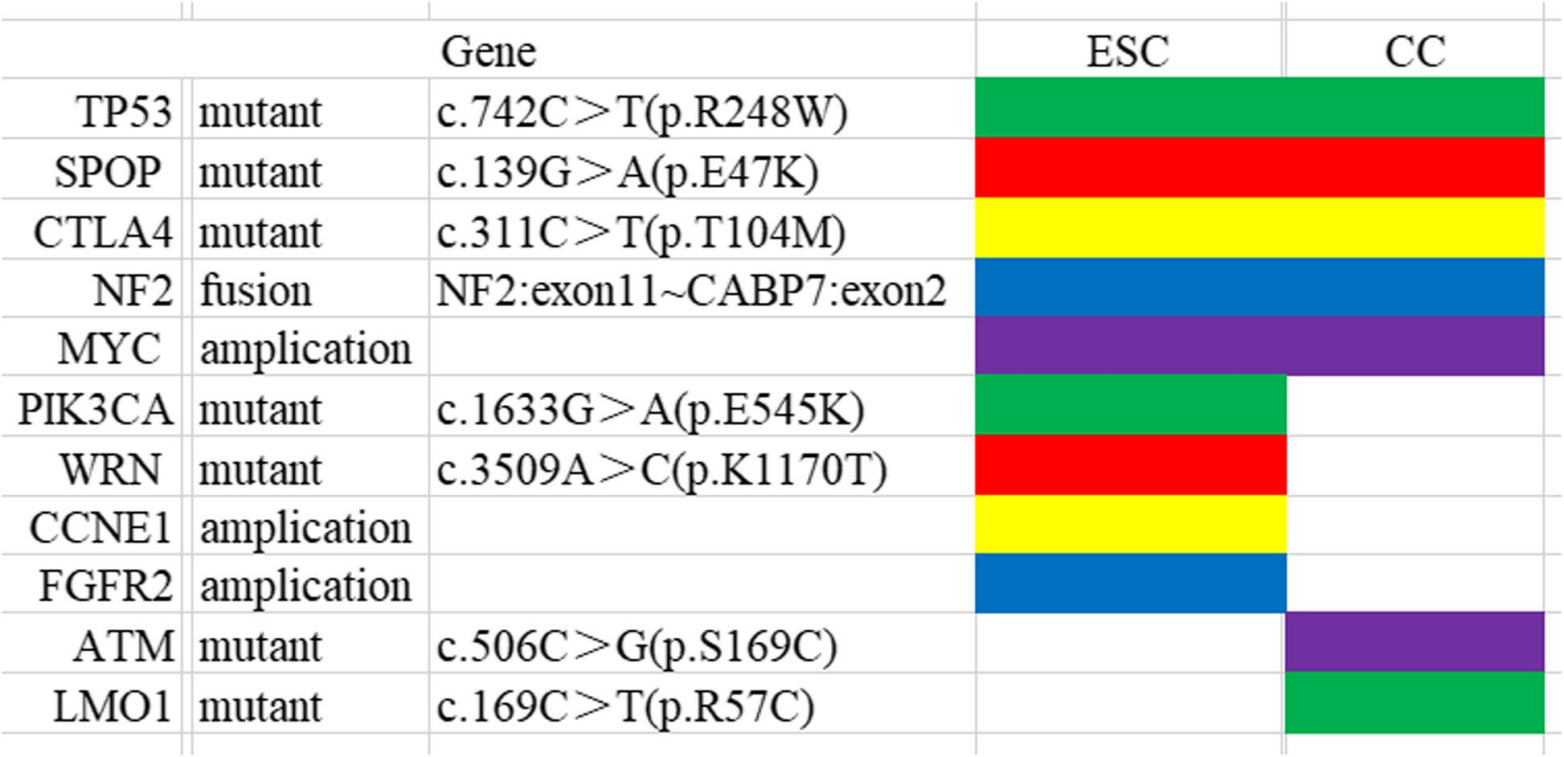
Common and difference of different gene changes in ESC with CC differentiation

Most genetic abnormalities were identical in both components including *TP53* c.742C > T (p.R248W), *SPOP* c.139G > A (p.E47K) and *CTLA4* c.311C > T (p.T104M) mutations, *MYC* amplification (copy number > 4), *NF2-CABP7* fusion (NF2: exon11 ~ CABP7: exon2 fusion), low tumor mutation burden (< 10/Mb), and microsatellite stability. Nevertheless, *ATM* c.506C > G (p.S169C) and *LMO1* c.169C > T (p.R57C) mutations were only found in CC while *PIK3CA* c.1633G > A (p.E545K), and *WRN* c.3509A > C (p.K1170T) mutations, *CCNE1* and *FGFR2* amplification were present in ESC (Fig. 4).

## Discussion and conclusion

Somatic choriocarcinoma is very uncommon in the female genital tract [2–10], prevailing in postmenopausal women. It also occurs in non-gynecologic organs, such as lung [11], breast [12], and gastrointestinal tract [13]. Somatic CC often concur with common carcinomas, mostly poorly differentiated adenocarcinomas. They have a very aggressive clinical course and adverse prognosis. Most patients died of disease within 12–18 months after surgery because of advanced tumor stage and lack of effective therapy. We reviewed 10 cases of somatic cancers with CC differentiation in female genital organs, predominantly in the ovary and uterus (Table1). Ten women were in advanced stage and 9 died of disease within one and a half year. Rawish et al. [10] reported that endometrial carcinoma with trophoblastic differentiation often followed an aggressive course with poor clinical outcomes.

**Table 1:** Epithelial malignancies with choriocarcinomatous differentiation in female genital organs

Case	age	organ	Final diagnosis	Stage surgery	FIGO stage	Follow up	reference
Case 1	50	Ovary	EAC + CC, SCC + CCC	TH-RSO, P LN;	IV	DOD 10 m	3
Case 2	48	ovary	CCC + CC	TH-BSO, OM, DO;	IV	DOD 11 m	4
Case 3	59	uterus	EAC + CC	TH-BSO, PLN	IIIC2	NED 2 m	5
Case 4	54	uterus	EC, G1 + CC	TH-BSO, SCR,	III	DOD 15 m	6
Case 5	61	uterus	ESC + CC	TH-BSO, PLN,	IV	DOD 2 m	7
Case 6	42	uterus	EAC + CC	TH-BSO, OM, AP, PLN	IB	NED 6 m	8
Case 7	34	uterus	Carcinosarcoma + CC	TH-BSO, OM, PLN	IV	DOD7m	9
Case 8	72	uterus	DEC + CC	TH-BSO, PLN, OM	IIIA	DOD7m	10
Case 9	77	uterus	EC, G1 + CC	TH-BSO, PLN, OM	IIIC2	DOD11m	
Case 10	62	uterus	DEC + CC	TH-BSO, PLN	IIIC1	DOD16m	
Case 11	68	uterus	ESC + CC	TH-BSO, MB, PLN	IIIA	DOD13m	Our case

*EAC* Endometrial adenocarcinoma, *CCC* Clear cell carcinoma, *SCC* Small cell carcinoma, *ESC* Endometrail serous carcinoma, *DEC* Dedifferentiated enodmetrial carcinoma, *CC* Choriocarcinoma, *EC* Endometrioid carcinoma, *G1* Grade 1, *TH-BSO* Total hysterectomy with bilateral salpingo-oophorectomy, *MB* Multiple biopsies, *TH-RSO* Total hysterectomy with right salpingo-oophorectomy, *PLN* Pelvic lymphadenectomy, *AP* Appendectomy, *OM* Omentectomy, *SCR* Sigmoid colon resection, *DO* Debulking operation, *DOD* Dead of disease, *NED* No evidence of the disease

The overlapping molecular change in ESC and CC components suggested their common clonal origin. However, such molecular tests of somatic choriocarcinoma were rarely performed. A case study analyzed the copy number variation profiles of chromosomes on the whole genomic found the same clonal origin between endometrioid carcinoma and carcinoma with trophoblastic differentiation separately [6]. Recently, Xing et al.[14] analyzed that choriocarcinomatous components of mixed gynecologic tumor was somatic origin. Except *PIK3CA* c.1633G > A (p.E545K), most mutations in our case were not detected in the case series of Xing et al. [14]. The morphology, immunohistochemistry and NGS test suggest that our case be categorized into the TCGA molecular subtype of copy number-high/TP53 mutant (CN-H; serous-like) endometrial carcinoma. However, no serous carcinoma was found by Xing [14]. The distinct subtype in our case is the fundamental cause that is responsible for the substantial molecular difference between that report and the present study.

*TP53* mutation is well documented in the pathogenesis of many cancers, and might be an indicator for poor prognosis, and chemoresistance [15]. *MYC* amplification can promote cell proliferation, immortalization, dedifferentiation and transformation in the initiation and development of tumors [16]. *CCNE1* amplification and *SPOP* mutation were two preferential genetic alterations in serous carcinoma [17] although the latter was more frequent in clear cell carcinoma than in serous carcinoma [18]. Neron et al. [19] found that *FGFR2* mutation was one of the driver mutations in endometrial carcinoma metastasis by analyzing the biological and genomic profiles in 11 pairs of primary and metastatic carcinomas. These genetic changes provide potential molecular biomarkers to uncover the poor prognosis of our case.

*CTLA4* is a key molecule in the regulation of immune checkpoint, and was mutated in ESC and CC. Moreover, we observed that PD-L1 was strong expressed in the CC component consistent with a previous report by Lu et al. [20]. These findings implicated a potential therapeutical benefit of immune checkpoint inhibition for our patient. Immune checkpoint inhibitors (ICI) targeting PD-1/PD-L1 have shown its promising anti-tumoral effects on various gynecologic cancers [21]. Unfortunately, our patient did not receive immunotherapy owing to its unavailability of the drugs at that time.

Choriocarcinomatous components of mixed gynecologic tumor was considered as “retrodifferentiation”, by which rejuvenation to early embryonic development may occur when differentiated cells lose their specific properties acquired during previous steps of mutations [14]. *LMO1* mutation is critical for cancer initiation and progression [22]. *ATM* mutation was germline mutations of non-Lynch syndrome genes which may be associated with endometrial cancer [23]. *LMO1* and *ATM* mutation in our CC also provided the evidence to support that CC might be dedifferentiated from ESC, thereby having a more dismal clinical outcome. Ashton [6] reported that carcinoma with trophoblastic differentiation was more aggressive than the endometrial carcinoma. We assume that epithelial malignancy with CC differentiation can be interpreted as a dedifferentiated form with a worse clinical outcome.

In summary, we describe a case of ESC with CC. NGS study provided evidence that CC was a retrodifferentiation form of ESC, as clonality evidence between the two components. The epithelial malignancy with CC differentiation was considered as a dedifferentiated tumor with rapid progression and poor prognosis.
